# Primary diffuse large B cell lymphoma of the prostate in a patient with HIV infection

**DOI:** 10.1002/iju5.12541

**Published:** 2022-10-05

**Authors:** Takashi Ujiie, Taketo Kawai, Tomoyuki Kaneko, Tadashi Yamamoto, Yasutoshi Oshima, Mutsuo Fujikura, Nobu Akiyama, Yuko Sasajima, Haruko Tashiro, Tohru Nakagawa

**Affiliations:** ^1^ Department of Urology Teikyo University School of Medicine Tokyo Japan; ^2^ Department of Internal Medicine Teikyo University School of Medicine Tokyo Japan; ^3^ Department of Pathology Teikyo University School of Medicine Tokyo Japan

**Keywords:** diffuse large B‐cell lymphoma, HIV, malignant lymphoma, prostate

## Abstract

**Introduction:**

Primary prostate lymphomas are very rare; however, the incidence of malignant lymphoma is high among HIV‐infected patients. Herein, we report a case of primary diffuse large B‐cell lymphoma (DLBCL) of the prostate in an HIV‐infected patient.

**Case presentation:**

A 47‐year‐old man presented with miction pain and back pain. Abdominal CT revealed a huge prostate mass extending to the left retroperitoneum. Serum sIL‐2R level was abnormally high (2896 U/mL), whereas PSA level was normal. HIV antigen and antibody tests were positive. The patient was diagnosed with DLBCL after a prostate biopsy. Systemic treatments were administered; however, the tumor was refractory, and the patient died 9 months after diagnosis.

**Conclusion:**

Prostate malignant lymphomas are rare but should be considered in patients with enlarged prostates and normal PSA levels. It should be noted that HIV patients have a high incidence of malignant lymphomas.

**Abbreviations & Acronyms**
CNS: central nervous system
CT: computed tomography
DLBCL: diffuse large B‐cell lymphoma
EBV: Epstein–Barr virus
HIV: human immunodeficiency virus
PSA: prostate‐specific antigen
R‐CHOP: rituximab, cyclophosphamide, doxorubicin hydrochloride, vincristine sulfate, and prednisone
R‐ESHAP: rituximab, etoposide, methylprednisolone, high dose cytarabine, and cisplatin
R‐GCD: rituximab, gemcitabine, carboplatin, and dexamethasone
sIL‐2R: soluble interleukin‐2 receptor

**Keynote message**

We report a case of primary diffuse large B‐cell lymphoma of the prostate in an HIV‐infected patient. Prostate malignant lymphomas are rare but should be considered in patients with normal PSA levels who have an abnormally enlarged prostate. Moreover, it should be noted that the incidence of malignant lymphoma is high among HIV patients.

## Introduction

Malignant lymphomas involving the prostate, especially primary prostate lymphomas that belong to lymphomas originating from extranodal sites, are extremely rare.^1^ Primary prostate lymphomas account for only 0.09% of all prostate malignancies and 0.1% of all non‐Hodgkin's lymphomas.^2^ However, the risk of non‐Hodgkin's lymphomas is very high in HIV patients.^3^ There are very few previous reports of primary prostate lymphomas in HIV‐infected patients. Herein, we report a case of primary diffuse large B‐cell lymphoma (DLBCL) of the prostate in an HIV‐infected patient.

## Case presentation

**Fig. 1:**
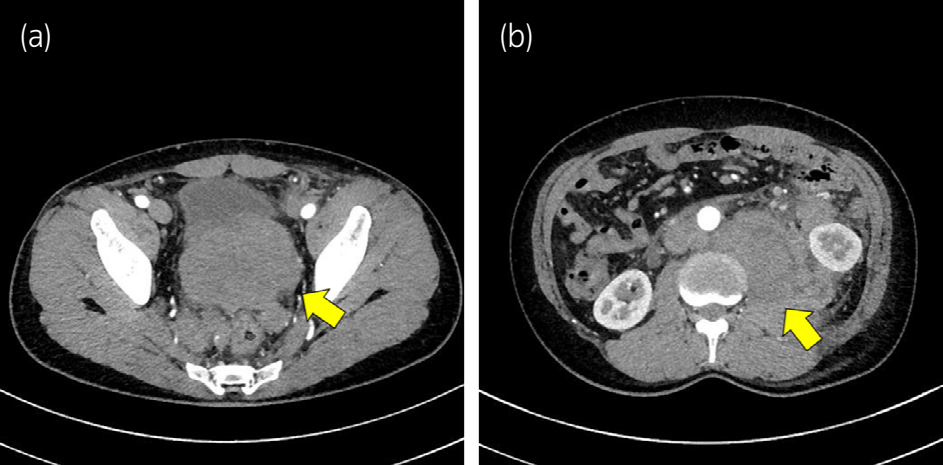
Abdominal CT findings. A huge prostate mass (a) with a volume of 220 mL extending to the left retroperitoneum (b).

A 47‐year‐old man visited our hospital due to 1‐month history of miction pain and back pain. Digital rectal examination revealed a goose‐egg‐sized, elastic, and hard prostate. Abdominal CT revealed a huge prostate mass (Fig. 1a) with a volume of 220 mL extending to the left retroperitoneum (Fig. 1b). Serum sIL‐2R, lactate dehydrogenase, and neuron‐specific enolase levels were abnormally high (2896 U/mL, 833 U/L, and 42.6 ng/mL, respectively), whereas the patient's PSA level was normal at 1.065 ng/mL. HIV antigen and antibody tests were positive. A western blot test confirmed a positive HIV diagnosis. The patient was a man who had sex with men, but this was the first indication of HIV infection and he had no subjective symptoms such as fever. No cytopenia was observed (white blood cell, 6700/μL; hemoglobin, 12.9 g/dL; platelet, 29.1 × 10^4^/μL). Laboratory examination revealed HIV‐1 RNA of 1.9 × 10^5^ copies/mL and CD4+ count of 58/μL.

**Fig. 2:**
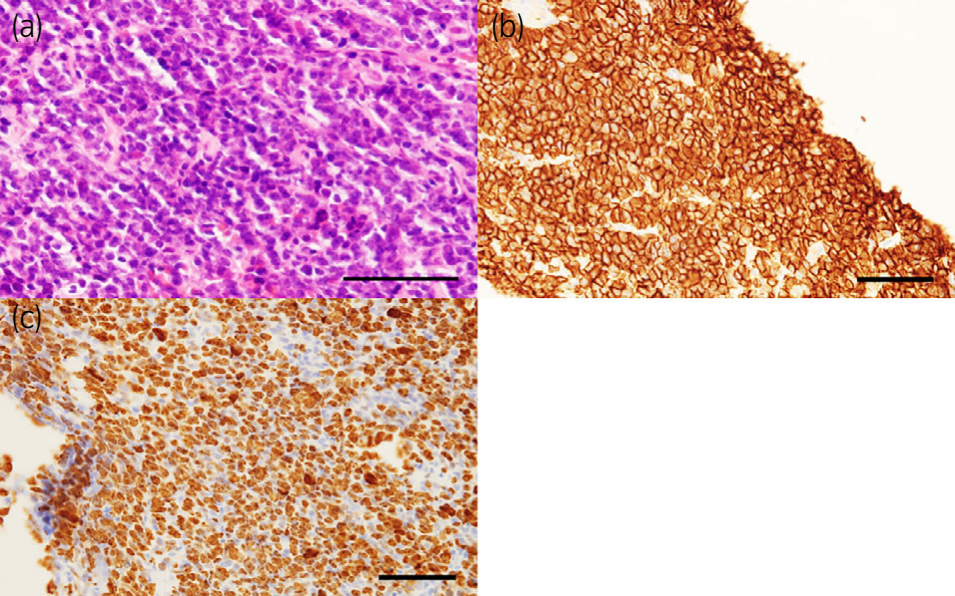
Histopathological findings. A diffuse proliferation of large lymphoid cells was observed using hematoxylin and eosin staining (a). Immunohistochemistry showed positive staining using CD20 (b). The Ki‐67 proliferation index was more than 80% (c). All bars indicate 50 μm.

Based on imaging findings and blood tests, we suspected malignant lymphoma of the prostate or atypical prostate cancer, such as small cell carcinoma. The patient underwent prostate biopsy 3 days after the first visit, and was diagnosed with DLBCL based on the following histopathological findings: a diffuse proliferation of large lymphoid cells was observed using hematoxylin and eosin staining (Fig. 2a), and immunohistochemistry showed positive staining for CD20 (Fig. 2b). The Ki‐67 proliferation index was more than 80% (Fig. 2c). Epstein–Barr virus‐encoded RNAs in situ hybridization was negative.

Two weeks after diagnosis, the patient complained of increasing back pain and abdominal pain. Whole‐body CT was performed, which revealed rapid growth of lymphoma in the prostate and left retroperitoneum and development of left hydronephrosis and a colovesical fistula. Head and spine MRI revealed no intracranial or intraspinal lesions, respectively. The patient underwent left ureteral stenting and urethral catheterization. Serum sIL‐2R level increased to 4383 U/mL. R‐CHOP was administered as systemic chemotherapy immediately, and lesions of lymphoma had almost disappeared after two courses. Serum sIL‐2R level decreased to 1161 U/mL. Tenofovir alafenamide/emtricitabine and dolutegravir were administered for HIV infection, resulting in undetectable HIV‐1 RNA after 4 months. The patient had frequent febrile urinary tract infections during myelosuppression due to colovesical fistulas, but colostomy could not be performed because of prolonged low CD4 levels.

After three courses of R‐CHOP, CT showed re‐enlargement of lymphoma in the prostate and right common iliac region. Serum sIL‐2R level re‐increased to 1883 U/mL. R‐ESHAP was administered 6 months after the diagnosis; however, after two courses, CT showed further enlargement of lymphoma in the prostate and right common iliac region and splenomegaly, and serum sIL‐2R level increased to 2765 U/mL. Seven months after diagnosis, one course of R‐GCD was administered. Eight months after the diagnosis, the patient was brought to the emergency room with impaired consciousness. Head CT revealed swelling, edema, and sulcal narrowing in the cerebral cortex, suggesting DLBCL infiltration of the central nervous system (CNS). In addition, abdominal CT showed invasion of the pelvic lesion into the sigmoid colon and rectum. He died 9 months after the diagnosis.

## Discussion

DLBCL is a type of lymphoma in which B cells with large cell nuclei proliferate diffusely. It is the most common and accounts for about 30% of non‐Hodgkin's lymphomas.^4^, ^5^ Approximately 30% of DLBCLs develop from extranodal organs, most commonly from the gastrointestinal tract.^5^ Two or more extranodal lesions and CNS involvement are poor‐prognostic factors.^6^

Malignant lymphomas originating from the prostate gland are rare. Most of the previously reported cases were DLBCL subtypes.^7^, ^8^ Lower urinary tract symptoms often appear, whereas specific symptoms related to lymphomas, such as fever, weight loss, and night sweats, rarely occur in the early stages.^7^, ^9^ PSA levels are usually normal or slightly high, which helps differentiate such lymphomas from prostate cancer.^9^, ^10^ Immunohistochemistry on biopsy also plays an important role in the diagnosis of prostate malignant lymphoma. The three criteria for the diagnosis of primary prostate lymphoma are (i) presence of symptoms due to prostate enlargement; (ii) predominance of prostate involvement with involvement elsewhere; and (iii) no liver, spleen, lymph node, or peripheral blood lesions within 1 month of diagnosis.^11^ The present case meets all of these criteria.

The risk of non‐Hodgkin's lymphoma is approximately 60–200 times higher in HIV patients than in the general population.^3^, ^12^, ^13^ DLBCL is the most common subtype, and the infection of Epstein–Barr virus (EBV) is often associated with it. Similar to other immunosuppression‐related lymphomas, lymphomas in HIV patients tend to involve extranodal sites, including CNS, gastrointestinal tract, liver, and bone marrow. The present case meets the following two criteria: the onset of extranodal lesions and CNS involvement, although histopathological findings were not suggestive of EBV infection.

To our knowledge, only one case of primary prostate lymphoma in an HIV‐infected individual has been reported.^1^ The report described a case of primary prostate lymphoma in a 26‐year‐old HIV‐positive male patient who was treated with 8 cycles of adriamycin‐based chemotherapy and radiotherapy to the prostate and achieved complete remission. The present report is the second reported case to describe primary prostate lymphoma in an HIV‐infected person.

## Conclusion

Prostate malignant lymphomas are rare but should be considered in patients with abnormally enlarged prostates and normal PSA levels. Moreover, it should be noted that HIV‐infected patients have a high incidence of malignant lymphoma.

## Author contributions

Takashi Ujiie: Data curation; writing – original draft. Taketo Kawai: Conceptualization; data curation; writing – original draft; writing – review and editing. Tomoyuki Kaneko: Supervision; writing – review and editing. Tadashi Yamamoto: Writing – review and editing. Yasutoshi Oshima: Data curation; writing – review and editing. Mutsuo Fujikura: Data curation; writing – review and editing. Nobu Akiyama: Writing – review and editing. Yuko Sasajima: Data curation; supervision; writing – review and editing. Haruko Tashiro: Supervision; writing – review and editing. Tohru Nakagawa: Conceptualization; project administration; supervision; writing – review and editing.

## Conflict of interest

The authors have no conflicts of interest to declare.

## Approval of the research protocol by an Institutional Reviewer Board

Not applicable.

## Informed consent

Written informed consent was obtained from the patient for publication of the details of this medical case and any accompanying images.

## Registry and the Registration No. of the study/trial

Not applicable.
